# Predicting mortality in the very old: a machine learning analysis on claims data

**DOI:** 10.1038/s41598-022-21373-3

**Published:** 2022-10-19

**Authors:** Aleksander Krasowski, Joachim Krois, Adelheid Kuhlmey, Hendrik Meyer-Lueckel, Falk Schwendicke

**Affiliations:** 1 Department of Oral Diagnostics, Digital Health and Health Services Research, Charité-Universitätsmedizin Berlin, Aßmannshauser Str. 4-6, 14197 Berlin, Germany; 2 Department of Restorative, Preventive and Pediatric Dentistry, Zmk Bern, University of Bern, Bern, Switzerland; 3 Institute of Medical Sociology and Rehabilitation Science, Charité-Universitätsmedizin, Berlin, Germany

**Keywords:** Geriatrics, Health services

## Abstract

Machine learning (ML) may be used to predict mortality. We used claims data from one large German insurer to develop and test differently complex ML prediction models, comparing them for their (balanced) accuracy, but also the importance of different predictors, the relevance of the follow-up period before death (i.e. the amount of accumulated data) and the time distance of the data used for prediction and death. A sample of 373,077 insured very old, aged 75 years or above, living in the Northeast of Germany in 2012 was drawn and followed over 6 years. Our outcome was whether an individual died in one of the years of interest (2013–2017) or not; the primary metric was (balanced) accuracy in a hold-out test dataset. From the 86,326 potential variables, we used the 30 most important ones for modeling. We trained a total of 45 model combinations: (1) Three different ML models were used; logistic regression (LR), random forest (RF), extreme gradient boosting (XGB); (2) Different periods of follow-up were employed for training; 1–5 years; (3) Different time distances between data used for prediction and the time of the event (death/survival) were set; 0–4 years. The mortality rate was 9.15% in mean per year. The models showed (balanced) accuracy between 65 and 93%. A longer follow-up period showed limited to no advantage, but models with short time distance from the event were more accurate than models trained on more distant data. RF and XGB were more accurate than LR. For RF and XGB sensitivity and specificity were similar, while for LR sensitivity was significantly lower than specificity. For all three models, the positive-predictive-value was below 62% (and even dropped to below 20% for longer time distances from death), while the negative-predictive-value significantly exceeded 90% for all analyses. The utilization of and costs for emergency transport as well as emergency and any hospital visits as well as the utilization of conventional outpatient care and laboratory services were consistently found most relevant for predicting mortality. All models showed useful accuracies, and more complex models showed advantages. The variables employed for prediction were consistent across models and with medical reasoning. Identifying individuals at risk could assist tailored decision-making and interventions.

## Introduction

In an ageing population, predicting mortality of elderly individuals is relevant, as such prediction may help to address possible sources of mortality, tailor an individual’s end-of-life management or plan healthcare resources appropriately. A wide range of prediction models in healthcare have been developed, most of them predicting other disease states than death or predicting death from a specific disease^1–3^. Often, explicit models, to be applied by a medical practitioner using a limited set of social and clinical covariates, have been developed^4^; such models are not able to reflect on the wide range of possible predictors available. Many of these models are also not necessarily applicable to the elder, non-hospitalized population, or are developed from small datasets, yielding limited stability. Most also use simple logistic regression (LR) models to make predictions.

More advanced machine learning (ML) like random forests (RF) or (extreme) gradient boosting algorithms ((X)GB) have been suggested to yield higher accuracy, especially when trained on complex and big data (e.g., claims data, which harbors a wide range of, at first glance, not necessarily relevant covariates), mainly as they are able to more appropriately reflect the complex internal structure of the data^5^. However, a range of studies showed that advanced ML offered only minimal improvements over LR to predict events like death^6^. On a large claims dataset involving 2.8 million individuals, for instance, LR was similar or even more accurate than advanced ML both short- and long-term^7^. A systematic review, focusing on palliative care, yielded a more nuanced picture, with advanced ML models outperforming LR to predict death when a sufficiently large and broad dataset was used to train the models, while using only basic administrative data did not allow the more advanced models to leverage their power. Notably, this review included only 3 studies comparing advanced ML and LR^8^. Another review corroborates these ambiguous findings, showing that advanced ML models were, in some cases, more accurate than LR for predicting myocardial infarction and associated mortality, but that even if such advantage was found, the magnitude of it was limited^9^.

In the present study, we used claims data from one large insurer, mainly acting in the Northeast of Germany, to develop and test differently complex ML prediction models, comparing them for their (balanced) accuracy, but also the importance of different predictors (i.e., the consistency of how these models came to their prediction). We further aimed to assess if the period of how long individuals were followed-up before the prediction was made (i.e. the amount of data accumulated and used for training) or the time between the data acquisition (exposure) and the outcome (death) impacted on prediction (balanced) accuracy. While claims suffer from a range of limitations like selection, confounding or misclassification bias, they yield large datasets (where advanced ML may be advantageous) on groups which are otherwise hard to assess (like the old, the sick, poor and rural living ones) with limited risks of reporting bias^10,11^.

## Methods

### Study design

The investigated cohort was evaluated based on claims data from one large statutory (public) health insurance in Germany. Old individuals (75 years or older) from the AOK Nordost were followed over 6 years (2012–2017). The AOK Nordost is the Northeastern regional branch of national insurer, the Allgemeine Ortskrankenkasse (AOK), active mainly in the federal states of Berlin, Brandenburg and Mecklenburg-Vorpommern. ML models of different complexity, notably LR, RF and XGB were used to predict our primary outcome, death in one of the years 2013–2017. The reporting of this study follows the RECORD statement^12^.

### Setting

The AOK Nordost insures around 1.8 million individuals mainly in the German capital, Berlin, and two rural states, Brandenburg and Mecklenburg-Vorpommern, with only few larger cities (> 70,000 inhabitants). Data for this study was routinely collected and provided under ethical approval in a pseudonymized form using a data protection cleared platform via the scientific institute of the AOK Nordost, the GEWiNO.

### Participants and sample size

A comprehensive sample of 404,610 statutorily insured very old, aged 75 years or above, living in the Northeast of Germany in 2012 was followed over the 6 years observational period. Hence, no formal sample size estimation was performed. Our feature extraction process needed a patient to survive for at least 1 year in order to make a prediction. Out of the total sample, 373,077 survived at least 1 year. Predicting their deaths was our target variable (outcome, see below).

**Figure 1:**
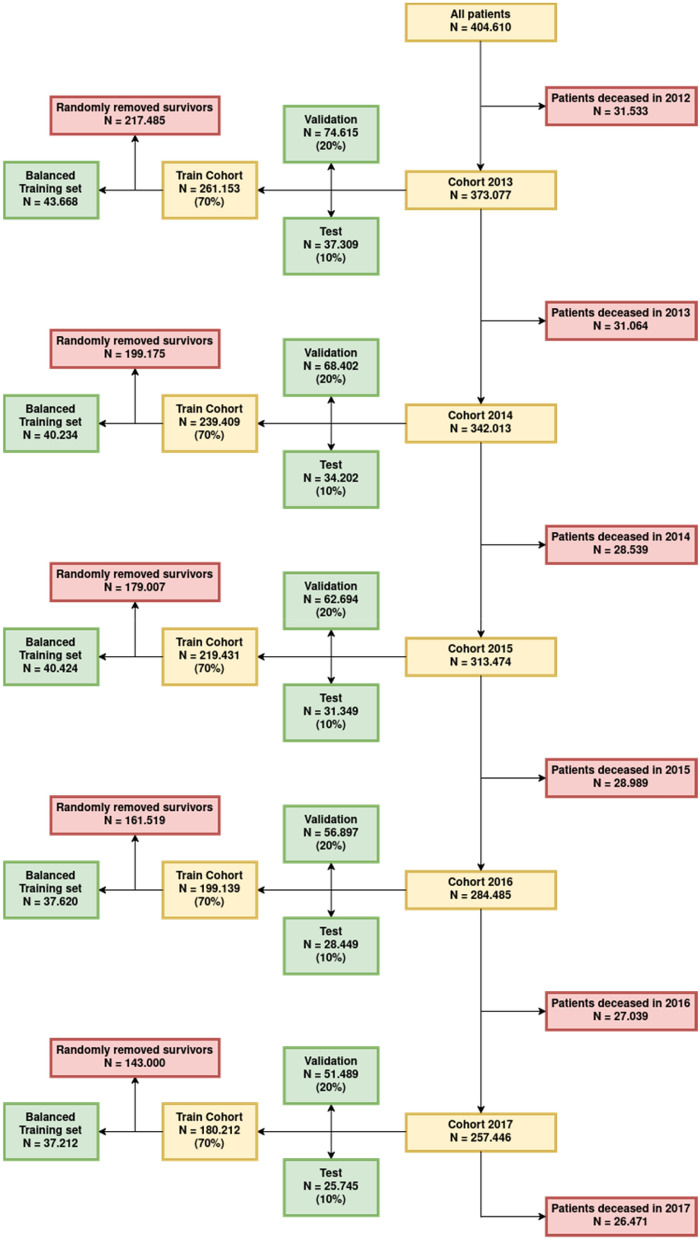
Flow of the data. Each combination of follow-up and time distance allowed for different datasets. The dataset used for training a model contained the maximum number of patients available who survived follow-up + time–distance respectively up to the target year. Training datasets have been created by randomly removing surviving patients such that their sample size matched that of deceased individuals. Positive target variable values were made up from patients dying during the following year. Validation and test datasets consisted of 20% and 10% of samples, including the respective amount of target values, correctly resembling the underlying population.

The dataset was imbalanced towards survival (89.7–91.7% of individuals survived in the years 2013–2017, respectively). To reduce bias in the model by predicting only the majority class (survival), a balanced training dataset was constructed by randomly choosing the same number of survivors as deceased patients (which reduced the training dataset, yet remaining considerably large, see Fig. 1). Validation- and test-sets remained imbalanced, resembling the mortality rate at given years in order to evaluate the models on a correct representation of the underlying population. Variable ascertainment was only possible via insurance base data and claims data. The database had been curated for plausibility by the GEWiNO.

### Variables

Our outcome was whether an individual died in the inspected year (2013–2017) or not. Our primary outcome metric was balanced accuracy^13^, i.e. accuracy where weighting samples in proportion to the sample size of given class results reduced class imbalance; secondary metrics were F1-score, sensitivity, specificity, and positive or negative predictive values (PPV/NPV).

The original dataset contained variables such as utilized services, costs and metadata like age and sex for each individual. These were either static (immutable by time) such as sex, or dynamic such as age, and were either given for a full calendar year such as annual total costs or given for an exact date each such as received treatment. Date variables were combined into intervals of 365.25 days, being counted backwards from death or from a random point in the follow-up period in the case of survival.

Overall, 86,326 potential variables were available in the dataset, the highest number coming from the following categories: 41,276 unique prescribed drugs; 20,149 unique operations or procedures and 22,913 unique diagnoses. To reduce dimensionality and complexity^14^ we chose to extract the 30 most important variables. Our feature extraction process followed an iterative process (Fig. S1): (1) We first chose the 140 variables which clinical experts found most promising to predict death. On these, feature importance was assessed first using Lasso Regression, a Linear Regression model with L1-Regularization, i.e. a term penalizing high coefficients. The Lasso Regression removes less useful features, by setting their coefficients to zero, to minimize the L1 term. By increasing the regularization value iteratively, forcing the Lasso Regression to drop more features, it is possible to create an order of importance of respective features before passing them on to a more complex model. Afterwards, Random Forest Classification models were trained using all features and iteratively excluding features with lesser importance until the classifiers (balanced) accuracy drastically decreased. (2) Then, we repeated that process by pooling the variables extracted in step (1) and all others except prescribed drugs, unique operations or procedures and diagnoses. We then performed another Lasso Regression and Random Forest Classification to exclude variables once more until the classifiers (balanced) accuracy decreased. (3) Afterwards we pooled variables extracted before with prescribed drugs, unique operations or procedures and diagnoses and repeated the exclusion step once more. Finally, the 30 features with highest Gini importance, i.e. features responsible for best splits and high probability of being reached in respective decision trees, in the Random Forest Classifier have been extracted. Appendix Table S1 summarizes all retained features, and Appendix Fig. S1 describes the variable extraction process.

### Follow-up and time distance

Data was available for years in between 2012 and 2017. In order to evaluate performance gains with an increasing follow-up period, i.e. sampling data from a bigger range of years (e.g. collecting data from 3 instead of 2 years for prediction making) and performance losses with predicting death further into the future (time distance until potential death, e.g. using data from up to 2015 versus data from up to 2017 to predict death in 2017, resulting in a time distance of 2); a time distance of 0 would result in predicting the 1-year mortality. We constructed 15 different datasets containing all possible combinations of follow-up periods (1–5 years) and time distances until death (0–4 years).

### Data sources and access

As described before^15,16^, data used for this study was provided by the GEWiNO using a data protection approved platform. Data were pseudonymized and included the described covariates among further variables. Comparability of data between different years and data consistency was given.

### Bias

Participants and providers were not aware that the claims data will be used for data analyses. Selection bias within this study was impossible for the target population (very old individuals insured at AOK Nordost). Although it is noteworthy that the cohort included in the dataset differs from the overall population of very old Germans and likely suffers from biases of claims data, as discussed later.

### Statistical analyses

The following ML models were employed: LR, a model assigning a probability for a target variable, which in our case was death, using a linear combination of available variables^17^. We trained multiple models for each dataset candidate, the only hyperparameter being the number of iterations which ranged between 2^3^ to 2^16^ in steps of powers of two. For training the L-BFGS optimization method was utilized^18^. All models converged before reaching 2^16^ iterations. The model with the highest (balanced) accuracy on the validation set was chosen. For 4 out of our 15 datasets this was not the model which converged on the training set.

RF, an ensemble model containing multiple decision trees classifying inputs into an arbitrary number of classes^19^. In our case the decision was binary, i.e. an individual survived or died. For Hyperparameter tuning, Random Search was utilized, in this case it consisted of randomly choosing 1000 candidate parameter combinations, namely number of estimators, i.e. decision trees, (5–1000); maximum number of random samples per decision tree used during training (1 − N); minimum number of samples in leaf nodes (1 − N/10), with N being the number of samples in the given training dataset. Each combination was trained independently and evaluated on the validation set. The RF with the combination of hyperparameters yielding highest (balanced) validation accuracy was chosen respectively.

XGB, an advanced ensemble machine learning model also utilizing decision trees, but unlike RF constructing them additively, not independently^20^. Hyperparameter tuning was equivalent to RF but with different parameters, namely number of estimators (5–1000); maximum decision tree depth (1–50); minimal child weight, i.e. minimal summed weight needed to construct a child node (1–20); gamma, i.e. minimum loss reduction needed in order to partition a node (0–2 in 0.1 steps); maximum number of bins, i.e. maximum number of distinct value groups per feature 2^8^–2^16^. The classifier with the combination of hyperparameters yielding highest (balanced) validation accuracy was chosen.

All analyses, modeling and visualization were performed using Python (version 3.6.12, available at http://www.python.org) and auxiliary modules, notably scikit-learn (version 0.24.1) and XGBoost (version 1.4.0).

### Ethical approval and informed consent

All experiments were carried out in accordance with relevant guidelines and regulations. Data collection was ethically approved by the ethics committee of the AOK Nordost. Informed consent was waived by the ethics committee of the of the AOK Nordost.

## Results

The flow of data is summarized in Fig. 1. As described, the sampled cohort included 373,077 individuals, 142,102 did not survive follow-up; characteristics of the cohort are shown in Table 1. The mean follow-up was 1816 days (standard deviation SD: 574). The mean (SD) age of the sample was 83.5 (5.1) years. The majority of individuals were female and younger than 85 years. The mortality rate was 9.15% in mean per year and higher in males than females and those with social hardship status than those without. Mortality increased incrementally and monotonically with increasing age; there were minor differences between federal states only.

**Table 1:** Sample characteristics (N; %) from Northeast Germany. Total, male and female population aged 75 years or older, in 5-years age bands and according to federal state. We compare the global dataset against the sampled cohort.

Covariate	Group	N	Mortality rate (%)	Mean annual total costs in EUR (years alive–year of death)	Mean annual transportation costs in EUR (years alive–year of death)
All		373,077 (100%)	9.15	9958 (7642–15,664)	340 (229–613)
Sex	Male	123,537 (33.1%)	10.02	9434 (6795–15,200)	376 (232–691)
	Female	249,540 (66.9%)	8.74	10,213 (8032–15,920)	322 (227–570)
Age group	75–79	155,671 (22.8%)	4.45	7267 (5221–15,127)	247 (151–619)
	80–84	258,718 (37.9%)	6.47	9221 (7252–15,940)	327 (228–666)
	85–89	166,720 (24.4%)	10.74	11,394 (9243–15,559)	400 (283–627)
	90–94	76,656 (11.2%)	17.90	13,877 (12,049–15,757)	435 (323–550)
	95–99	20,587 (3.0%)	26.92	15,896 (15,551–16,127)	429 (345–485)
	100–104	3584 (0.5%)	37.88	16,191 (17,806–15,728)	353 (288–371)
	105–109	318 (0.05%)	44.83	16,162 (20,307–15,055)	268 (264–269)
Social hardship status	No	191,018 (51.2%)	8.47	6704 (4956–11,720)	201 (129–409)
	Yes	182,059 (48.8%)	9.87	13,374 (10,719–19,002)	485 (343–786)
Federal state	Berlin	112,359 (30.1%)	9.25	10,057 (7594–16,105)	269 (177–496)
	Brandenburg	140,912 (37.8%)	8.95	9550 (7305–15,284)	364 (244–653)
	Mecklenburg	99,041 (26.6%)	9.07	9851 (7583–15,545)	359 (241–653)
	Others*	20,531 (5.5%)	10.54	12,918 (10,987–16,317)	472 (358–677)

*“Others” indicates individuals who were insured at AOK Nordost, but did not live in the three federal states of interest.

**Figure 2:**
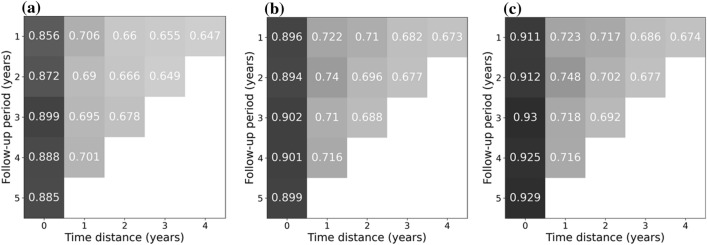
Balanced accuracy of predicting death using Logistic Regression (**a**), Random Forests (**b**) and Extreme Gradient Boosting (**c**). Models built on different follow-up periods (from 1 to 5 years) in which covariates may have occurred, and used different time distances (time between exposure and event [death]).

The models showed useful (balanced) accuracy between 65 and 93% (Fig. 2). A longer follow-up period showed limited to no advantage in predicting death, but models trained on data with short time distance from death were more accurate than models trained on more distant data. RF and XGB were more accurate than LR for every follow-up and time distance. The difference between RF and XGB, however, was limited, and the possible advantage of XGB was only apparent when having data available up until death.

There was consistency in the most important variables employed for prediction by different models: The utilization of laboratory diagnostic services and transportation costs (per year and in 365 days intervals) as well as the consumption of conventional (not urgent) outpatient care were relevant for all three models; utilization of an emergency transport was relevant for LR and XGB (Table 2). For LR, the direction of associations was notable; having had emergency hospital visits and transports as well as any hospital visits significantly increased the risk of dying, while having had laboratory analytics and having consumed conventional outpatient care decreased it.

**Table 2:** The most important predictors used by logistic regression (LR), random forest (RF) and extreme gradient boosting (XGB). For LR, Odds Ratios and 95% confidence intervals (95% CI; from 100 independently trained LR models) are shown to display variable importance, for RF and XGB Gini importance is displayed. Models represented are from dataset with 1 year of follow-up and no time distance to the point of death.

LR	Odds ratio	Lower CI	Upper CI
Emergency hospital visits [count]	1.379	1.357	1.401
Claimed non-emergency (conventional) outpatient services [count]	0.689	0.687	0.692
Hospital visits [count]	1.270	1.257	1.284
Claimed laboratory analytics [count]	0.733	0.729	0.738
Emergency transport utilized [yes]	1.245	1.228	1.263

RF	Gini importance (%)	XGB	Gini importance (%)
Total transportation cost in 365 days intervals	36.0	Emergency transport utilized [yes]	44.8
Claimed non-emergency (conventional) outpatient services [count]	25.36	Claimed non-emergency (conventional) outpatient services [count]	11.2
Transportation cost billed per calendar year	6.2	Total transportation cost in 365 days intervals	8.6
Insurance points claimed	5.9	Claimed laboratory analytics [count]	3.9
Total costs billed per calendar year	4.3	Transportation cost billed per calendar year	2.9

**Figure 3:**
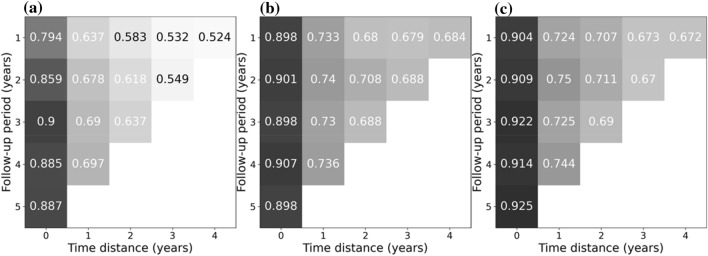
Sensitivity for predicting death using Logistic Regression (**a**), Random Forests (**b**) and Extreme Gradient Boosting (**c**). Models built on different follow-up periods (from 1 to 5 years) in which covariates may have occurred, and used different time distances (time between exposure and event [death]).

**Figure 4:**
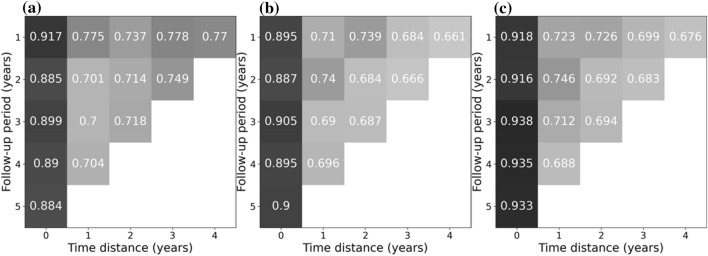
Specificity for predicting death using Logistic regression (**a**), Random forests (**b**) and Extreme Gradient Boosting (**c**). Models built on different follow-up periods (from 1 to 5 years) in which covariates may have occurred, and used different time distances (time between exposure and event [death]).

All secondary metrics (F1-score, sensitivity, specificity, PPV and NPV) showed similar behavior as (balanced) accuracy (in Table 3, details for exemplary models built on 1-year follow-up and time distances between 0 and 4 years are shown). Notably, for RF and XGB sensitivity and specificity were similar, while for LR sensitivity was significantly lower than specificity (Figs. 3 and 4). For all three models, the NPV significantly exceeded 90% for all analyses, while the PPV was below 62% (and even dropped to below 20% for longer time distances from death, see Table S2).

**Table 3:** Metrics for predicting death using logistic regression (a), random forests (b) and extreme gradient boosting (c). Models built on a follow-up period of 1 year and time distances (from 0 to 4 years).

Time distance [years]	Balanced accuracy	F1-score (weighted)	Sensitivity	Specificity	PPV	NPV
**(a)**						
0	0.856	0.918	0.794	0.917	0.453	0.981
1	0.706	0.812	0.637	0.775	0.205	0.959
2	0.66	0.778	0.583	0.737	0.184	0.946
3	0.655	0.797	0.532	0.778	0.204	0.94
4	0.647	0.788	0.524	0.77	0.206	0.934
**(b)**						
0	0.896	0.911	0.898	0.895	0.426	0.99
1	0.722	0.776	0.733	0.71	0.187	0.967
2	0.71	0.787	0.68	0.739	0.209	0.958
3	0.682	0.748	0.679	0.684	0.187	0.952
4	0.673	0.73	0.684	0.661	0.187	0.948
**(c)**						
0	0.911	0.928	0.904	0.918	0.488	0.991
1	0.723	0.783	0.724	0.723	0.192	0.966
2	0.717	0.781	0.707	0.726	0.208	0.961
3	0.686	0.757	0.673	0.699	0.193	0.952
4	0.674	0.739	0.672	0.676	0.192	0.948

## Discussion

Predicting death on claims data has a range of relevant applications, for example early and targeted intervention by healthcare professionals, or risk-adjusted insurance policies and incentives. So far, few studies have used large claims dataset and analyzed them using ML for predicting mortality^21–23^. In the present evaluation, data from over 370,000 elderlies from a large area in Germany were used to train and test ML models; the underlying dataset contained over 86,000 features. We aimed to employ ML for predicting and to also assess if the models build their predictions in a consistent manner and according to clinical reasoning.

We found (balanced) accuracies which ranged between 65 to 93%, i.e. moderately useful to excellent, depending on three factors (ordered according to their relevance); time distance to death; ML model architecture; follow-up period (i.e. number of accumulated data a model was trained on). A range of predictors were assessed and found both consistently across models and in line with clinical reasoning. Notably, while the (balanced) accuracies were rather high (as were the NPVs), the positive predictive values were low. This and more details need to be discussed.

First, the time distance to an event (death or survival) was most relevant for (balanced) accuracy. It was apparent that events close to death were features the models built on. Notably, these were rather indicators of poor health (e.g. requiring an emergency transport, costs for transportation) but not specific health conditions or risk factors (which were available in the dataset, but not found that relevant for prediction making). Vice versa, we did not find benefits in increasing the follow-up period, i.e. in expanding the dataset available for training, which is likely a function of the relevance of distance to death: Data acquired 3 or 4 years ago was too distant and hence not useful for prediction making any longer. If claims data are to be used for prediction making, it seems relevant to record and employ them in a timely manner, i.e. to avoid lengthy delays in documentation, records cleaning and making them available for modelling.

Second, ensemble models showed advantages over the less complex LR. This has been found before^24^ and was, to some degree expected given the size of our dataset. While in smaller datasets, more complex models oftentimes do not yield advantages or even show limitations due to overfitting (i.e. learning the training data by heart and not generalizing sufficiently any longer), in large datasets they can exert their power and yield superior accuracies. Notably, the interpretation of these models is no longer possible for humans, which is why it was relevant to contrast the feature importance (Gini importance) of RF and XGB with the most impactful coefficients of LR (Odds-Ratio furthest away from 1), where we can assess the direction of any underlying association, for instance.

Third, we found these associations and the feature importance to be both consistent across models and clinically meaningful. Certain factors (the need of and costs for transportation being first and foremost, followed by the need for an emergency and, more generally, any hospital visit) increased the risk of death in LR, while others (receiving conventional outpatient care, laboratory analytics) decreased it. While the former indicates, as discussed, the poor health of people before dying, the latter can be considered as indicators of moderate to good health (e.g. as individuals were able to receive outpatient care on their own).

Last, sensitivity, specificity and NPV were high or very high (NPV exceeding 98% means that nearly every individual identified to be not at risk eventually survived—this certainty is most relevant for any application in the practice or insurance setting). On the other hand, the PPV was very low; individuals identified to be at risk were, as indicated above, by no means not at high risk of dying. This low PPV and high NPV were mainly the result of the low incidence of death (9% per year). With this regard, models with a high sensitivity are especially desired, another reason why LR does not seem the best choice when implementing any such model into a healthcare application.

This study has a number of strengths and limitations. First, it is one of few longitudinal investigations in the very old and comprises a cohort of over 370,000 individuals from three different federal states. Second, a massive variety in covariates could be employed and considered during feature extraction, many of them at first glance not directly related to mortality. This, however, is one of the promises of big data and advanced analytics; identifying complex patterns in data beyond the capacity of humans and building highly accurate models on that. Third, as indicated, further refinement of the developed models may assist in implementing a useful application in the insurance or practice setting: Data owners like insurers or practices could regularly feed models with claims data to identify insurance holders or patients at risk. These could, for example, be scheduled for more regular check-ups, which could be additionally incentivized, or could be offered specific preventive or monitoring programs. Admittedly, identifying individuals at risk comes with significant ethical conflicts (e.g. individuals have a “right not to know”) and moral hazards (e.g. risk-based insurance premiums). Fourth, and as a limitation, claims data suffer from a range of biases. For example, we cannot easily infer from claimed to provided or even needed treatments. Certain possibly relevant factors (e.g. care status) were not available and accounted for, and some available factors (e.g. social hardship status, place of living) came with limited granularity. Fifth, individuals insured by AOK Nordost are not fully representative for all Germans, as they are less affluent and tend to be older than the national average. The rural–urban disparities are also more severe in this region, with Berlin as capital and some of the poorest and most rural German municipalities being spatial neighbors. Moreover, privately insured individuals are not at all reflected; these are usually more affluent given the entry barrier to private insurance being a minimum salary (or being self-employed or a public servant). Notably, their share is very low in this part of Germany. Last, in order to avoid bias coming from oversampling techniques, we instead opted for undersampling, which itself may introduce bias. Notably, undersampling was conducted for training, not testing, hence reflecting the true value of the models for predicting death in the full population.

In conclusion, all ML models showed useful accuracies to predict mortality, but more complex models showed advantages over LR. Making predictions far into the future was less accurate than more short-term predictions. The variables employed for prediction were consistent across models and with medical reasoning. Identifying individuals at risk could assist tailored decision-making in daily care and allow targeted interventions on individual and insurance level.

## Supplementary Information

Supplementary Information.

## Data Availability

Data used in this study cannot be made available by the authors given data protection rules, but may be requested at the GEWiNO.
