# Supplementary material for: Predicting mortality in the very old: a machine learning analysis on claims data
Source: Sci Rep. 2022 Oct 19;12:17464. doi: 10.1038/s41598-022-21373-3 (PMC9581892; doi:10.1038/s41598-022-21373-3)
Supplement: Supplementary file 1 — Supplementary Information. [file 41598_2022_21373_MOESM1_ESM.docx]

**Supplementary material**


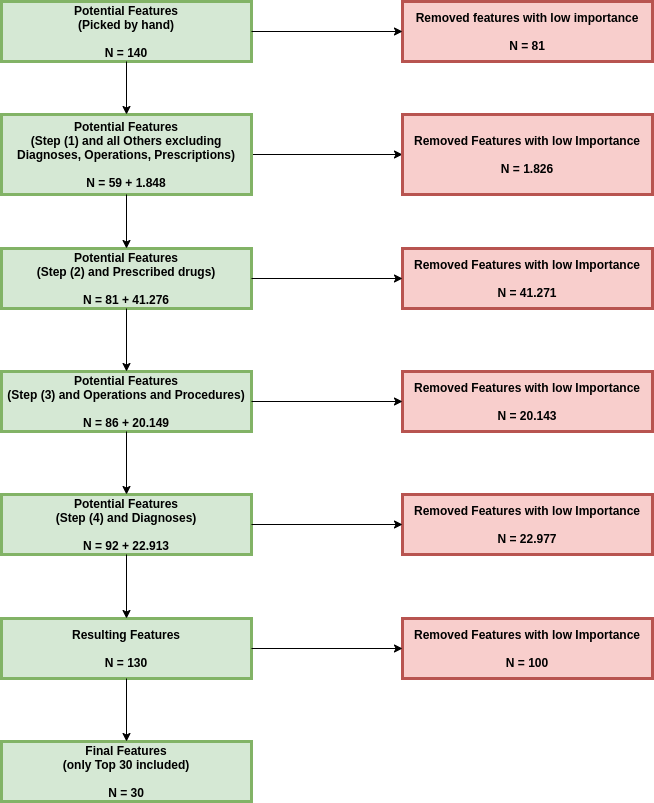


Figure S1: Iterative extraction of 30 most important features using Lasso Regression and Random Forest Classifiers.

Table S1: Features retained and used for modelling.

| Feature | Type |
| --- | --- |
| Claimed services by several providers jointly | numerical (count) |
| Claimed services during the night | numerical (count) |
| Claimed conventional outpatient services | numerical (count) |
| Claimed services by a deputy provider | numerical (count) |
| Claimed laboratory services | numerical (count) |
| Ambulant insurance service points | numerical (sum) |
| Prescriptions from general physician | numerical (count) |
| Novaminsulfon prescriptions | numerical (count) |
| Pantoprazole prescriptions | numerical (count) |
| Ramilich prescriptions | numerical (count) |
| Spironolactone prescriptions | numerical (count) |
| Torasemide prescriptions | numerical (count) |
| Valsatran prescriptions | numerical (count) |
| Prescriptions costs | numerical (sum, 365 day intervals) |
| Transportation costs | numerical (sum, 365 day intervals) |
| Emergency transport utilized [True] | binary |
| Age | numerical |
| Sex [female] | binary |
| Social hardship status [True] | binary |
| General hospital vists | numerical (count) |
| Emergency hospital visits | numerical (count) |
| Dentist costs | numerical (sum, per calendar year) |
| Drug costs | numerical (sum, per calendar year) |
| Hospital costs | numerical (sum, per calendar year) |
| Transportation costs | numerical (sum, per calendar year) |
| Remedies costs | numerical (sum, per calendar year) |
| Aiding tools costs | numerical (sum, per calendar year) |
| Home care costs | numerical (sum, per calendar year) |
| Total costs | numerical (sum, per calendar year) |
| Rehabilitation costs | numerical (sum, per calendar year) |

Table S2: Metrics for predicting death using logistic regression (a), random forests (b) and extreme gradient boosting (c). Models built on different follow-up periods (from 1-5 years) in which covariates may have occurred, and used different time distances (time between exposure and event [death]).

| Follow-up [years] | Time distance [years] | Balanced accuracy | F1-Score | Sensitivity | Specificity | PPV | NPV |
| --- | --- | --- | --- | --- | --- | --- | --- |
| 1 | 0 | 0.856 | 0.918 | 0.794 | 0.917 | 0.453 | 0.981 |
| 2 | 0 | 0.872 | 0.901 | 0.859 | 0.885 | 0.408 | 0.986 |
| 3 | 0 | 0.899 | 0.912 | 0.9 | 0.899 | 0.476 | 0.989 |
| 4 | 0 | 0.888 | 0.904 | 0.885 | 0.89 | 0.466 | 0.986 |
| 5 | 0 | 0.885 | 0.899 | 0.887 | 0.884 | 0.466 | 0.986 |
| 1 | 1 | 0.706 | 0.812 | 0.637 | 0.775 | 0.205 | 0.959 |
| 2 | 1 | 0.69 | 0.761 | 0.678 | 0.701 | 0.186 | 0.956 |
| 3 | 1 | 0.695 | 0.759 | 0.69 | 0.7 | 0.199 | 0.954 |
| 4 | 1 | 0.701 | 0.759 | 0.697 | 0.704 | 0.216 | 0.952 |
| 1 | 2 | 0.66 | 0.778 | 0.583 | 0.737 | 0.184 | 0.946 |
| 2 | 2 | 0.666 | 0.763 | 0.618 | 0.714 | 0.189 | 0.946 |
| 3 | 2 | 0.678 | 0.764 | 0.637 | 0.718 | 0.209 | 0.944 |
| 1 | 3 | 0.655 | 0.797 | 0.532 | 0.778 | 0.204 | 0.94 |
| 2 | 3 | 0.649 | 0.776 | 0.549 | 0.749 | 0.2 | 0.935 |
| 1 | 4 | 0.647 | 0.788 | 0.524 | 0.77 | 0.206 | 0.934 |

| Follow-up [years] | Time distance [years] | Balanced accuracy | F1-Score | Sensitivity | Specificity | PPV | NPV |
| --- | --- | --- | --- | --- | --- | --- | --- |
| 1 | 0 | 0.896 | 0.911 | 0.898 | 0.895 | 0.426 | 0.99 |
| 2 | 0 | 0.894 | 0.905 | 0.901 | 0.887 | 0.423 | 0.99 |
| 3 | 0 | 0.902 | 0.917 | 0.898 | 0.905 | 0.492 | 0.989 |
| 4 | 0 | 0.901 | 0.91 | 0.907 | 0.895 | 0.484 | 0.989 |
| 5 | 0 | 0.899 | 0.912 | 0.898 | 0.9 | 0.508 | 0.987 |
| 1 | 1 | 0.722 | 0.776 | 0.733 | 0.71 | 0.187 | 0.967 |
| 2 | 1 | 0.74 | 0.792 | 0.74 | 0.74 | 0.223 | 0.966 |
| 3 | 1 | 0.71 | 0.756 | 0.73 | 0.69 | 0.203 | 0.959 |
| 4 | 1 | 0.716 | 0.757 | 0.736 | 0.696 | 0.22 | 0.958 |
| 1 | 2 | 0.71 | 0.787 | 0.68 | 0.739 | 0.209 | 0.958 |
| 2 | 2 | 0.696 | 0.749 | 0.708 | 0.684 | 0.194 | 0.956 |
| 3 | 2 | 0.688 | 0.747 | 0.688 | 0.687 | 0.204 | 0.95 |
| 1 | 3 | 0.682 | 0.748 | 0.679 | 0.684 | 0.187 | 0.952 |
| 2 | 3 | 0.677 | 0.733 | 0.688 | 0.666 | 0.191 | 0.949 |
| 1 | 4 | 0.673 | 0.73 | 0.684 | 0.661 | 0.187 | 0.948 |

| Follow-up [years] | Time distance [years] | Balanced accuracy | F1-Score | Sensitivity | Specificity | PPV | NPV |
| --- | --- | --- | --- | --- | --- | --- | --- |
| 1 | 0 | 0.911 | 0.928 | 0.904 | 0.918 | 0.488 | 0.991 |
| 2 | 0 | 0.912 | 0.926 | 0.909 | 0.916 | 0.498 | 0.991 |
| 3 | 0 | 0.93 | 0.942 | 0.922 | 0.938 | 0.602 | 0.992 |
| 4 | 0 | 0.925 | 0.939 | 0.914 | 0.935 | 0.603 | 0.99 |
| 5 | 0 | 0.929 | 0.938 | 0.925 | 0.933 | 0.611 | 0.991 |
| 1 | 1 | 0.723 | 0.783 | 0.724 | 0.723 | 0.192 | 0.966 |
| 2 | 1 | 0.748 | 0.798 | 0.75 | 0.746 | 0.229 | 0.967 |
| 3 | 1 | 0.718 | 0.77 | 0.725 | 0.712 | 0.213 | 0.96 |
| 4 | 1 | 0.716 | 0.753 | 0.744 | 0.688 | 0.218 | 0.958 |
| 1 | 2 | 0.717 | 0.781 | 0.707 | 0.726 | 0.208 | 0.961 |
| 2 | 2 | 0.702 | 0.756 | 0.711 | 0.692 | 0.199 | 0.957 |
| 3 | 2 | 0.692 | 0.752 | 0.69 | 0.694 | 0.208 | 0.95 |
| 1 | 3 | 0.686 | 0.757 | 0.673 | 0.699 | 0.193 | 0.952 |
| 2 | 3 | 0.677 | 0.743 | 0.67 | 0.683 | 0.195 | 0.948 |
| 1 | 4 | 0.674 | 0.739 | 0.672 | 0.676 | 0.192 | 0.948 |
